# The Influence of Facial Asymmetry on Genuineness Judgment

**DOI:** 10.3389/fpsyg.2021.727446

**Published:** 2021-11-25

**Authors:** Bérénice Delor, Fabien D’Hondt, Pierre Philippot

**Affiliations:** ^1^Louvain Experimental Psychopathology, Psychological Sciences Research Institute, Catholic University of Louvain (UCLouvain), Louvain-la-Neuve, Belgium; ^2^Inserm, CHU Lille, U1172-LilNCog-Lille Neuroscience and Cognition, Université de Lille, Lille, France; ^3^Clinique de Psychiatrie, Unité CURE, CHU Lille, Lille, France; ^4^Centre national de ressources et de résilience Lille-Paris (CN2R), Lille, France

**Keywords:** facial asymmetry, facial expression, emotional facial expression, genuineness, decoding

## Abstract

This study investigates how asymmetry, expressed emotion, and sex of the expresser impact the perception of emotional facial expressions (EFEs) in terms of perceived genuineness. Thirty-five undergraduate women completed a task using chimeric stimuli with artificial human faces. They were required to judge whether the expressed emotion was genuinely felt. The results revealed that (a) symmetrical faces are judged as more genuine than asymmetrical faces and (b) EFEs’ decoding is modulated by complex interplays between emotion and sex of the expresser.

## Introduction

The face and its various emotional expressions provide many information about the expresser’s state and characteristics that are important for social interactions (Ross et al., 2007). Among other, facial expressions constitute a critical non-verbal component to judge whether an expressed emotion is genuinely felt or posed in the absence of the corresponding emotional state (Dawel et al., 2017). However, an event-elicited and genuinely expressed emotion does not guarantee that the Emotional Facial Expression (EFE) will be judged as genuine by the perceiver (Dawel et al., 2017). Zloteanu and Krumhuber (2021, p.4) referred to a “demeanor bias”: although the emotion and intent are genuinely expressed, other factors can influence the perceiver’s judgment in the sense of fakeness. The determinants of perceived genuineness, however, are still relatively unexplored in the literature.

Genuineness judgment would mostly rely on appearance-based cues rather than explicit knowledge about the expresser’s behavior or personality (Rudoy and Paller, 2009). Some aspects of EFEs are thus directly relevant for genuineness processing. A likely candidate is the symmetry of the EFE. EFE asymmetry refers to one side of the face expressing a different emotion than the other side (Ekman and Friesen, 1975; Ekman et al., 1990). The most common example is the smirk. In contrast to the symmetrical smile suggesting a real enjoyment, the smirk suggests either the presence of a withheld and more genuine emotional state or an “experience of two competing primary emotions during a social situation” (Ross et al., 2013, p. 253). Facial asymmetry can also appear because the two sides of the face express the same emotion but at different intensities. For instance, electromyography studies showed that left hemiface muscles are more expressive than right muscles, whether the methodology used posed or spontaneous, positive or negative EFEs (for a review, see Powell and Schirillo, 2009). However, although EFEs in natural settings are predominantly asymmetrical (for a review, see Borod et al., 1997), this issue has received poor empirical consideration and the research has mainly focused on facial asymmetry during emotional expression rather than visual perception. Previous research (e.g., Rhodes, 2006; Sofer et al., 2014) suggests that the distance from the subjective prototype of a specific social category is an indicator of fakeness. As prototypes are derived from the “mathematic average trait values” for a category, EFEs prototypes are likely to be symmetrical. Symmetrical EFEs would therefore have a genuineness advantage over asymmetric EFEs because of a subjective experience of prototypically.

The nature of the expressed emotion might also be an important source of information for genuineness judgment. Facial expressions can encompass multiple social emotions that are not systemically genuine (Ekman and Friesen, 1975). Indeed, social emotions are inherently characterized by display rules, resulting from an “intensification, a minimization, a neutralization, a simulation, a dissimulation, or a qualification (facial blends of emotion) of the primary emotion” (Ross et al., 2013, p.253). In this vein, some authors conceptualize the function of facial expressions as 2-fold: either reflecting a genuine emotional state or communicating signals of affect and intent (Zloteanu and Krumhuber, p.2). The “demeanor bias” (Zloteanu and Krumhuber, 2021) could therefore be fostered when the perceiver guesses the social function of the EFE. Based on the well-known expressions of smirk (i.e., an asymmetrical smile that only involves the left or right lips) or the “Non-Duchenne” smile (i.e., a false smile that only involves the lower face; Ekman et al., 1990), participants might judge a facial expression of happiness as less genuine by suspecting social desirability intent. Expression of anger or fear might rather be judged as an event-elicited facial leakage (Ekman, 1997), as they are less likely to be motivated by social desirability.

The current study investigates how asymmetry and emotional display of the expresser might affect genuineness judgment by the perceiver (operationally defined as “whether the emotional expression displayed by another person is a genuine reflection of its underlying affect”; Zloteanu and Krumhuber, 2021, p.2). We specifically predict that individuals will judge (a) symmetrical faces as more genuine than asymmetrical faces due to a subjective gap with EFEs prototypes and (b) happy faces as less genuine than angry and fearful faces due to their socially conditioned nature. Those hypotheses will be investigated using male and female artificial human faces, allowing for the assessment of potential gender influences during genuineness judgment. Moreover, hemispheric lateralization during EFEs visual processing have been widely documented, showing that asymmetrical EFEs occurring in natural settings might be more (or less) easily processed according to the hemiface on which they appear. The right-hemisphere hypothesis has received strong empirical support (Borod et al., 1986; Christman and Hackworth, 1993; Workman et al., 2000; Asthana and Mandal, 2001; Nicholls et al., 2004; Alves et al., 2009). It states a right brain hemisphere specialization for EFEs processing regardless of the emotional valence, so that individuals would visually perceive more accurately EFEs appearing in their left visual field (Levy et al., 1983; Workman et al., 2000; Alves et al., 2009; Bear et al., 2016). Although hemispheric specialization does not constitute the focus of this study, asymmetry will be considered as a binary variable to explore the perceptual bias towards the left visual field.

## Materials and Methods

### Participants

Thirty-five undergraduate volunteers in psychology were recruited from the laboratory’s participant pool. Only women volunteered to be part of the project. The sample size was determined by conducting an *a priori* power analysis with G*Power [statistical test=ANOVA: repeated measures, within factors; effect size *f*=0.20; *α* error probability=0.05; power (1−*β* error probability)=0.80; correlation among repeated measures=0.7; Faul et al., 2007]. Participants ranged from 18 to 25 of age (mean=21.06, SD=1.999) and were native French speakers. Their participation was voluntary and paid 10 euros.

### Visual Stimuli

**Figure 1:**
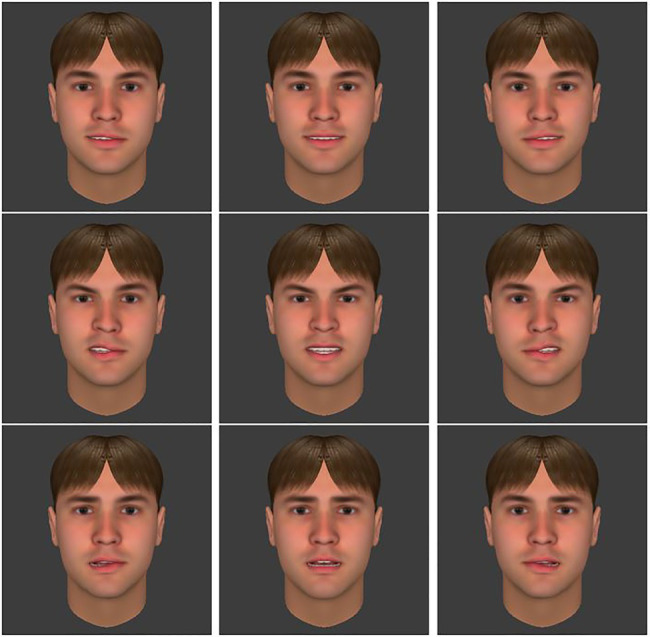
Illustration of the three (a)symmetries (respectively left hemiface, symmetry, and right hemiface) of each emotion (respectively happiness, anger, and fear) for the young man.

Visual stimuli were Caucasian artificial human faces designed with the FaceGen software (version 3.5.3). Chimeric faces (350×350 pixels) were created by splitting EFE pictures down the vertical midline and by recombining each half-face with a neutral half-face of the same identity. Artwork was used to ascertain that hair arrangements look natural. Three EFEs were used: happiness, anger, and fear, based on FaceGen parameter. As a whole, 36 visual stimuli were created: 4 (two males and two females)×3 (happiness, anger, and fear)×3 (symmetry, right hemiface, and left hemiface) (see examples in Figure 1). Stimuli are available from the authors on a simple request.

### Procedure

Emotion genuineness was conceptualized in this study as continuous rather than dichotomous (for evidence supporting genuineness being continuous, see Dawel et al., 2017). Participants had to judge the genuineness of the 36 visual stimuli on a five-point Likert scale (1=“Not at all genuine”; 5=“Totally genuine”), without time-limit and stimuli repetition. The scale included the clear meaning of each point (e.g., 3=“slightly genuine”). All instructions were given explicitly by the experimenter and then displayed on the computer screen. Participants were seated at 50 centimeters of a 17″ computer screen.

Procedure of this study was approved by the Ethical Committee of the Psychological Science Research Institute of the UCLouvain, and performed in accordance with the ethical standards as laid down in the 1964 Declaration of Helsinki and its later amendments or comparable ethical standards. Before testing, participants freely signed a written informed consent to participate in a study designed to explore the visual perception of emotional displays.

## Results

The statistical analyses were performed using IBM SPSS Statistics software (version 25). Individual means of Likert judgments for each EFE score were submitted to repeated-measures ANOVA with (a)symmetry (symmetry, left asymmetry, and right asymmetry)×emotion (happiness, anger, and fear)×sex (male and female) of expressers as within-subject factors. In addition to the main effects, interactions between variables were also investigated to explore the potential moderating role of each variable, hence suggesting insights for future research. Significant F-tests were followed up with pairwise *t*-tests adjusted with Bonferroni correction.

As shown in Table 1, the analyses revealed a strong main effect of (a)symmetry, emotion, and sex. Consistent with the prototypicality hypothesis, the main effect of (a)symmetry on genuineness judgment indicated that symmetrical EFEs were judged as being more genuine than asymmetrical EFEs, *F*(1.403,47.711)=66.381, *p*<0.001, *η*^2^*_p_*=0.661. The interaction (a)symmetry×emotion confirmed this pattern for all the three emotions, *F*(4,136)=2.455, *p*<0.049, *η*^2^*_p_*=0.67. Moreover, *post hoc* comparisons revealed no significant differences between faces with an EFE on the right hemiface and faces with EFE on the left hemiface, except for fear. In this latter case, asymmetrical EFEs were judged as more genuine, followed by fear faces with an EFE on the left hemiface, and then by fear faces with an EFE on the right hemiface.

**Table 1:** Means and SDs for genuineness (1=“Not at all genuine”; 5=“Totally Genuine”), as a function of (A)Symmetry, Emotion, and Sex.

(A)Symmetry	*F*(1.403,47.711)=66.381, *p*<0.001, *η*^2^*_p_*=0.661^**^		Symmetry	Right asymmetry	Left asymmetry
			4.210 (0.541)^a^	3.183 (0.659)^b^	3.291 (0.600)^b^
Emotion	*F*(2,68)=10.810, *p* <0.001, *η*^2^*_p_* =0.241^**^		Happiness	Anger	Fear
			3.795 (0.602)^a^	3.217 (0.717)^b^	3.719 (0.699)^a^
Sex	*F*(1,34)=18.517, *p* <0.001, *η*^2^*_p_* =0.353^**^		Female	Male	
			3.459 (0.502)^a^	3.695 (0.533)^b^	
(A)Symmetry×Emotion	*F*(4,136)=2.455, *p* <0.049, *η*^2^*_p_* =0.067^*^		Happiness	Anger	Fear
		Symmetry	4.343 (0.591)^a,i^	3.864 (1.044)^b,i^	4.457 (0.577)^a,i^
		Right Asymmetry	3.464 (0.796)^a,ii^	2.979 (0.834)^b,ii^	3.214 (1.011)^a,ii^
		Left Asymmetry	3.579 (0.740)^a,ii^	2.807 (0.868)^b,ii^	3.486 (0.874)^a,b,iii^
(A)Symmetry × Sex	*F*(2,68)=5.657, *p* =0.005, *η*^2^*_p_* =0.143^**^		Symmetry	Right Asymmetry	Left Asymmetry
		Female	4.195 (0.567)^a,i^	3.091 (0.701)^b,i^	3.091 (0.646)^b,i^
		Male	4.248 (0.604)^a,i^	3.348 (0.660)^b,ii^	3.491 (0.653)^b,ii^
Emotion × Sex	*F*(2,68)=24.730, *p* <0.001, *η*^2^*_p_* =0.421^**^		Happiness	Anger	Fear
		Female	3.481 (0.727)^a,i^	2.991 (0.735)^b,i^	3.905 (0.715)^c,i^
		Male	4.110 (0.634)^a,ii^	3.443 (0.789)^b,ii^	3.533 (0.827)^b,ii^

a, b, c *Indicate the (non) significant differences for horizontal lines*.

i, ii, iii *Indicate the (non) significant differences for vertical columns*.

* *Indicates p<0.05;*

** *Indicate p<0.01*.

The main effect of emotion showed that EFEs expressing happiness and fear were perceived as more genuine than those expressing anger, *F*(2,68)=10.810, *p*<0.001, *η*^2^*_p_*=0.241. This main effect was qualified by significant two-way interactions. The interaction (a)symmetry×emotion confirmed the main effect for symmetrical faces and faces with an EFE on the right hemiface. When the EFE is only on the left hemiface, fear did not differ significantly from happiness and anger, and happiness was the EFE judged to be the most genuine. Moreover, the interaction emotion×sex highlighted that female faces were indeed judged as more genuine when expressing fear, followed by happiness, with anger being judged the least genuine, *F*(2,68)=24.730, *p*<0.001, *η*^2^*_p_*=0.421. In contrast, male faces were judged as the more genuine when expressing happiness. Fear and anger were judged equivalently as less genuine.

Surprisingly, the main effect of expresser’s sex showed that male displays were judged as more genuine than female displays, *F*(1,34)=18.517, *p*<0.001, *η*^2^*_p_*=0.353. However, the interaction (a)symmetry×sex showed that this judgment favoring male faces appeared only in their asymmetrical configuration as no significant differences were found when appearing with symmetrical configuration, *F*(2,68)=5.657, *p*=0.005, *η*^2^*_p_*=0.143. Moreover, the interaction emotion×sex suggested that male faces were only judged as being more genuine than female faces for happy and angry faces. When expressing fear, female faces were significantly judged as more genuine than male faces.

## Discussion

In the present study, symmetrical EFEs were judged as more genuine than asymmetrical faces, with no perceptual differences between asymmetrical faces with an EFE in the left or the right visual field. Therefore, although the left visual field bias has not been demonstrated (Levy et al., 1983; Workman et al., 2000; Alves et al., 2009; Bear et al., 2016), the present study supports our hypothesis that the genuineness of a facial display is an inverse function of its subjective distance from the EFE prototype (Rhodes, 2006; Sofer et al., 2014). Indeed, prototypical facial expressions are always represented as symmetrical, although asymmetrical EFEs are more common than symmetrical expressions in everyday life (Rhodes, 2006; Alter and Oppenheimer, 2009; Sofer et al., 2014). Further, as prototypical EFEs are derived from the mathematic average of many EFE instances, their prototypes are likely to be symmetrical, lateral asymmetries compensating each other. Still, those results are preliminary and warrant future research to investigate whether symmetrical EFEs are indeed judged as more prototypical and whether prototypicality judgments correlate with genuineness judgments.

Results also showed that happy and fearful faces were judged as more genuine, which goes against the initial hypothesis opposing happiness to anger and fear. A possible explanation advocates a social advantage for EFEs encouraging prosocial intentions (Marsh et al., 2005; Dawel et al., 2017). Indeed, previous studies (e.g., Ekman and Friesen, 1975; Marsh et al., 2005, p.122) suggested that fearful EFEs promotes approach and helpful responses from the perceiver. In contrast to angry EFEs primarily perceived as aversive or threatening, fearful EFEs represent an “appeasement cue, intended to ameliorate conflict or elicit conciliatory or affiliative behavior by showing an affiliation desire or a submissive gesture” (Marsh et al., 2005, p.122). In this perspective, happy and fearful EFEs would be presumed as more likely to be genuine because observers might be more inclined to respond to an affiliative (versus threatening) intention (Dawel et al., 2017).

Interestingly, results also revealed that male faces were perceived as more genuine by the female participants when expressing happiness and anger (but not fear). This is inconsistent with previous studies attesting to an own-gender bias (participants of the present study were only women). This bias leads individuals to better recognize EFEs when appearing in a face of their own gender (e.g., Lovén et al., 2011). In the present study, the task was not to identify the emotional nature of the EFE but its genuineness. Participants’ judgment might have been influenced by the stereotype that males are more directly and less subtly expressive than females. Nevertheless, gender was investigated in this study in an exploratory way and in an all-women sample. Further evidence is required to draw strong conclusions on such a phenomenon. Especially, these stereotypes might be shared, different, or nonexistent among men.

This study also showed that female faces were judged as being more genuine when expressing fear while male faces were judged as more genuine when expressing happiness. These results are well accounted for by Becker et al. (2007) who asked participants to determine whether faces expressed anger, happiness, and fear, or no (neutral) expression. They showed that accuracy was higher when fear appeared on a female face rather than a male face. Again, these results suggest that gender stereotypes influence EFEs processing at least among women. Stereotypes would lead to cultural display rules (Ekman and Friesen, 1975) and reflect the personal and endorsed belief that men and women express emotions differently (Plant et al., 2000; Becker et al., 2007).

## Limitations of the Present Study and Perspectives

Some limitations have to be acknowledged. First, the artificial stimuli used in this study prevented methodological issues due to the use of real human faces (e.g., contrasts, luminosity, face orientation, structural differences between the two hemifaces, etc.; Kowner, 1995). However, those stimuli may suffer from a lack of ecological validity. Their artificial nature prevents analysis according to the posed (i.e., generated without being necessarily experienced authentically) or event-elicited nature of EFEs. Moreover, analyses of the present study were conducted without considering structural asymmetries that are specific to the human facial anatomy (e.g., scalp shape, wrinkles, and malformations), and without varying levels of asymmetry and averageness.

Second, the generalizability of the results is limited by the use of an all-women sample. Future studies should explore the interplay of the expresser and perceiver genders in the judgment of the genuineness of facial expressions. Although it has not been demonstrated in this study, there is empirical evidence for an own-gender bias during face recognition (e.g., Lovén et al., 2011). Nevertheless, its manifestation needs further consideration. On the one hand, some studies showed that only women manifested an own-gender bias (e.g., Lewin and Herlitz, 2002; Rehnman and Herlitz, 2006, 2007). On the other hand, Palmer et al. (2013) showed that the magnitude of the own-gender bias among women decreases when attentional resources are divided between two tasks.

## Conclusion

The present study focused on EFEs as it is a critical cue shaping daily interactions and social judgment. Results revealed that asymmetrical EFEs were judged as less genuine than symmetrical EFEs, an observation that had not been reported yet in the literature. Moreover, an advantage for EFEs fostering social interactions and affiliation also emerged. Finally, results supported that gender stereotypes influence the EFEs processing at least among women, advantaging fearful faces for female faces, and happy faces for male faces.

## Data Availability Statement

The raw data supporting the conclusions of this article will be made available by the authors, without undue reservation.

## Ethics Statement

The studies involving human participants were reviewed and approved by the Ethical Committee of the Psychological Science Research Institute of the UCLouvain. The patients/participants provided their written informed consent to participate in this study.

## Author Contributions

BD, FD, and PP contributed to conception and design of the study. BD organized the database, performed the statistical analysis, and wrote the first draft of the manuscript. All authors contributed to manuscript revision, read, and approved the submitted version.

## Funding

BD is funded by the “Fonds spéciaux de recherche (FSR)” (UCLouvain, Belgium). This Funding Agency did not exert any editorial influence or censorship on any part of this article.

## Conflict of Interest

The authors declare that the research was conducted in the absence of any commercial or financial relationships that could be construed as a potential conflict of interest.

## Publisher’s Note

All claims expressed in this article are solely those of the authors and do not necessarily represent those of their affiliated organizations, or those of the publisher, the editors and the reviewers. Any product that may be evaluated in this article, or claim that may be made by its manufacturer, is not guaranteed or endorsed by the publisher.
